# Relationship between different types of complement syntax and false belief in Mandarin-speaking children with autism spectrum disorder and typically developing children

**DOI:** 10.3389/fpsyg.2022.1045227

**Published:** 2022-11-21

**Authors:** Qiang Guo, Qianqian Pan, Qiaoyun Liu, Tingzhao Wang, Shuqin Cao, Yunqiang Lin, Bisheng Hu

**Affiliations:** ^1^College of Child Development and Education, Zhejiang Normal University, Hangzhou, China; ^2^National Institute of Education, Nanyang Technological University, Singapore, Singapore; ^3^Faculty of Education, East China Normal University, Shanghai, China; ^4^Department of Special Education, School of Education, Shaanxi Normal University, Xi’an, China; ^5^Zhejiang Bema Postdoctoral Workstation, Hanzhou, China

**Keywords:** complement syntax, autism spectrum disorder, Mandarin, false belief, theory of mind

## Abstract

Previous studies have shown that complement syntax is closely associated with false belief (FB) in children with autism spectrum disorder (ASD). However, the relationship between different types of complement syntax and FB remains unclear. This study examined the relationship between different types of complement syntax and FB in both ASD and typically developing (TD) children. Thirty Mandarin-speaking ASD and TD children, each matched for language ability, were included. Children completed different types of complement syntax tasks, verbal and nonverbal FB. For the ASD children, results demonstrated that sentential complement syntax independently predicted verbal and nonverbal FB, while phrasal complement syntax only predicted nonverbal FB. For the TD children group, sentential complement syntax only predicted verbal FB. This indicates that as the language demands of the FB task decrease, ASD children can use both types of complement syntax for its prediction. Moreover, the characteristics of ASD children differ from TD children in terms of the relationship between different types of complement syntax and FB. The results of this study support de Villiers’ point of view from the Mandarin perspective and provide evidence for the social-cognitive component of the theory of mind.

## Introduction

Theory of mind plays an important role in interpersonal interactions, specifically regarding the ability to understand mental states (Happé et al., 1999). One classic paradigm is the False Belief (FB) task, which reflects children’s theory of mind through cognitive conflict tasks, such as the change-in-location task and the unexpected content task (Baron-Cohen et al., 1985; Perner et al., 1987). Typically developing (TD) children experience FB milestones around 4–5 years of age (Chen et al., 2005; Milligan et al., 2007). Subsequently, children with autism spectrum disorder (ASD) perform poorly on FB tasks (Leekam and Perner, 1991). Studies have shown that when the language difficulty of the FB task decreases, scores for ASD children improve, and differences between ASD and TD children are reduced (Su and Wang, 2004). Some researchers have noted that the complexity of language in the FB task may mask children’s true abilities (Chandler et al., 1989). For instance, some ASD individuals with better language skills can pass the FB task (Lind and Bowler, 2009). This indicates that language is an important factor affecting the comprehension of the FB task in ASD children (Tager-Flusberg and Joseph, 2005).

De Villiers and de Villiers (2000) argued that complement syntax is an important part of FB development. However, this concept has not been verified in different language systems (Cheung et al., 2004). Although there may be a correlation between complement syntax and FB, these relationships differ depending on the type of complement syntax and FB (Ng et al., 2010). Furthermore, meta-analyses have shown that complement syntax can help ASD children understand FB to some extent, but has no facilitative effect on TD children (Farrar et al., 2017). The current study aims to examine the following questions to build upon the existing literature.

Can complement syntax help Mandarin-speaking ASD children understand FB?

Is the relationship between complement syntax and FB affected by the type of complement syntax and the linguistic difficulty of the FB task?

Do ASD children and TD children share the same characteristics in that relationship?

To the best of our knowledge, this is the first study to explore the relationship between different types of complement syntax and FB in Mandarin-speaking children with ASD. Our findings help reveal the relationship between language and theory of mind of Mandarin-speaking ASD children and could provide insight into early intervention for FB in ASD children from a linguistic perspective.

### Complement syntax and verbal false belief

There is a close correlation between complement syntax and verbal FB. Through a one-year longitudinal study, de Villiers and Pyers (2002) found that complement syntax was the most powerful predictor of FB. Although the study provided evidence for a relationship between complement syntax and FB, it did not control for factors such as age and working memory. Mo and Su (2009) found that after controlling for age, working memory, and language comprehension, complement syntax still independently accounted for FB variance in Mandarin-speaking children. They also found that complement syntax cues in the FB task improved TD children’s scores (Mo et al., 2007). Although Cheung et al. (2004) did not find an independent predictive effect of complement syntax on FB after controlling for general language, the two were significantly correlated.

Experimental studies on ASD children have also supported the predictive effect of complement syntax on FB. Tager-Flusberg and Joseph (2005) found in a follow-up study of English-speaking ASD children that complement syntax from the first year predicted FB in the same year and the second year. This result has been validated in cross-linguistic studies, but cross-linguistic variability has also emerged. Lin et al. (2015) found that complement syntax with communicative or psychological verbs was able to predict FB in Chinese-speaking ASD children. Complement syntax with psychological verbs had a large explanatory effect on FB. In a study on French ASD children, although there was a significant correlation between complement syntax and FB, complement syntax did not predict verbal FB after controlling for general language (Durrleman et al., 2016). There are two potential explanations for these findings: first, there was significant heterogeneity in a small number of subjects included in the study; second, there may have been differences in the language systems. Therefore, it is necessary to explore these relationships in Mandarin-speaking ASD children further.

### Complement syntax and nonverbal false belief

As language complexity in the FB task may affect the relationship between complement syntax and FB, it is necessary to determine whether complement syntax could also be a significant predictor of FB in nonverbal tasks. Durrleman et al. (2016) showed that after controlling for morphosyntax, complement syntax was still able to explain nonverbal FB independently. Studies of children with hearing impairment and children with specific language impairment also provide evidence for the relationship between the two (de Villiers and de Villiers, 2012; Durrleman et al., 2017). However, some research has shown that complement syntax is not intrinsically related to nonverbal FB. Studies on the theory of mind in French-speaking ASD children have shown that while complement syntax was significantly biased in relation to verbal FB, it was not significantly correlated with non-verbal FB (Durrleman and Franck, 2015). Furthermore, no significant correlations were found between complement syntax and nonverbal FB in TD children (Wiesmann et al., 2016). Because of inconsistent research findings on the relationship between complement syntax and nonverbal FB, it is necessary to examine this relationship further. Further, few studies have examined the relationship between complement syntax and nonverbal FB in Mandarin-speaking populations, especially in ASD children.

### Different types of complement syntax and verbal false belief

The research described above focuses on the relationship between complement syntax, mostly the *that* (tenses) complement, and FB. In English, in addition to the “that” complement, there is also a *to* (infinitival) complement (Bloom et al., 1980; Tardif and Wellman, 2000). Cheung et al. (2004) explored the relationship between two types of complement syntax (*that* complement and *to* complement) and FB in English-speaking and Cantonese-speaking TD children and showed that the two types of complement syntax were only related to FB and could not predict FB independently. However, Cheung (2006) subsequent research failed to find a predictive effect for the two types of complement syntax on FB in Cantonese TD children. In contrast, Ng et al. (2010) found that the two types of complement syntax were significantly correlated with FB, but those relationships were influenced by the linguistic demands of the test task. This suggests that the relationship between different types of complement syntax and FB is heterogeneous, but not fully explored. Although Mandarin does not have explicit morphological markers or morphemes in grammatical morphology like English, there are clear distinctions in the components following the predicate verb in complement syntax, such as phrasal complement syntax (subject + predicate + verb phrase, *Xiaoming wangle na pingguo*) and sentential complement syntax (subject + predicate + clause, *Mama yiwei xiaoming shangxue qule*) (Lu, 2006; Wang, 2018). In terms of grammatical complexity, phrasal complement syntax is simpler and easier for children to master (Tardif and Wellman, 2000; Arndt, 2019). Few studies, however, have studied whether phrasal complement syntax can help children with ASD comprehend FB or whether sentential complement syntax plays the same role in FB prediction.

### Differences in false belief understanding between children with autism spectrum disorder and typically developing

There are different ways of representing the theory of mind between ASD and TD children (Low and Perner, 2012; Farrar et al., 2017). Tager-Flusberg and Sullivan (2000) point out that there are two components in processing theory of mind: social perceptual and social cognitive. The social perceptual component focuses on the attribution of information, such as voices, faces, or body gesture, and thus judge others’ mental state. The social cognitive component makes inferences about the mental states of others through cognitive abilities, such as language and working memory.

TD children understand FB through either the social perceptual or cognitive components, whereas ASD children have impairments in social perception and are unable to make effective attributions about others’ intentions and visual information (Baron-Cohen et al., 1997). Therefore, they cannot effectively understand FB through the social perceptual component, but they can integrate information with social cognitive components, such as language, to bypass impairment mechanisms and achieve a representation of FB (Tager-Flusberg and Joseph, 2005). Since the two-component theory of Tager-Flusberg and Sullivan (2000) is based on western culture, can this theory explain the representation of the theory of mind for Mandarin-speaking ASD children? If this theory has an explanatory role, is the representation of FB in ASD children consistent with that of TD children? These questions need to be further explored.

### The present study

This study explores the relationship between different types (sentential and phrasal) of complement syntax and FB in Mandarin-speaking ASD children, as well as the similarities and differences in the relationship between ASD and TD children. Based on the results of previous studies and the two-component theory proposed by Tager-Flusberg and Sullivan (2000), we hypothesized that:

1. Complement syntax could predict FB in Mandarin-speaking ASD children.
2. The predictive effects of sentential and phrasal complement syntax on FB would differ and would be influenced by the linguistic difficulty of the FB task.
3. Mandarin-speaking ASD and TD children would have different characteristics in the relationship between complement syntax and FB. ASD children would need to use language, such as complement syntax, to understand FB; however, TD children would have multiple representations.

## Materials and methods

### Participants

Participants were recruited from autism rehabilitation centers or special education schools in Zhuji, Xuzhou, and Tai’an (all three cities are in eastern China). Information was posted online or on-site to recruit autism participants. Participants were selected according to the following criteria: (1) diagnosis of ASD by clinicians according to the DSM-IV or DSM-V at tertiary hospitals; (2) a Childhood Autism Rating Scale (CARS) score greater than 30, scored by the investigators; (3) children’s daily expressed sentences of four Chinese characters or more, provided by parents; (4) a sentence comprehension correction rate of 50% or more, as measured by the sentence comprehension ability subtest of the Mandarin Clinical Evaluation of Language (Liu, 2021); (5) a non-verbal intelligence score of 65 or more on the Combined Raven’s Test (Li et al., 1988); and (6) daily communication in Mandarin between ASD children and their parents.

Thirty ASD children were included in the study (25 boys and 5 girls, age range 65–151 months, mean age 86.17 months^1^). The control group included TD children matched by language ability in ASD children. Thirty TD children were included in the study (15 boys and 15 girls, age range 45–100 months, mean age 63.97 months). Similar to the ASD children, TD children’s daily communication language was Mandarin, excluding other potential barriers, such as intellectual disability and hearing impairment. The TD children were recruited from an elementary school in Zhuji. The TD children were matched using raw scores from the Peabody Picture Vocabulary Test-Revised (PPVT-R) (Sang and Miao, 1990). The differences in PPVT - R raw scores between ASD and TD children were controlled by 5 points. There were no statistically significant differences in PPVT-R scores between TD and ASD children (*t* = 0.08, *p* > 0.05). This study was approved by the Human Ethics Committee of [the first author’s university]. Parents of the participants provided written informed consent for participation.

### Materials

#### Combined Raven’s test

The CRT is a non-verbal intelligence test with 72 questions divided into six units: A, Ab, B, C, D, and E. It was revised by Li et al. (1988). The CRT retest reliability is 0.95 with a correlation coefficient using the Wechsler Intelligence Scale for Children of 0.56. The test requires participants to complete questions by selecting the most appropriate pattern from the available options. There is no time limit on the test. Participants with autism must complete at least units A, Ab, and B. The test is stopped when 3 consecutive errors are made in units C, D, and E. One point is awarded for each correct response.

#### Peabody picture vocabulary test-revised

Based on Dunn’s revised version, the PPVT-R was revised by Sang and Miao (1990). It contains 175 questions and has good Chinese cultural applicability. PPVT-R has split-half reliability of 0.99, retest reliability of 0.94, and is widely used in autistic children (Ren and Sang, 2005). The PPVT-R was used to measure and match participants’ language abilities. The participants were asked to identify pictures by listening to words. The test was stopped when six out of eight consecutive questions were incorrect. The child was credited with one point for each correct answer.

#### Childhood autism rating scale

The CARS, developed by Schopler et al. (1980), is predominately used for diagnosing autism. The scale is divided into 15 sub-items and scored using a 5-point Likert rating scale. The CARS simply provided confirmation of the autism diagnosis in this study because the ASD children included in this study were required to have a diagnosis of autism from a tertiary hospital.

#### Sentence comprehension test

The sentence comprehension test was selected from the sentence comprehension subtests from the Mandarin Clinical Evaluation of Language, developed by Liu (2021). The test’s Cronbach reliability coefficient was 0.90. Since the test does not contain complement syntax, it was used as a sentence comprehension test. A sentence-listening-picture identification method was used in which participants selected the corresponding pictures based on the sentences they heard. One point was awarded for each correct response, and the test was stopped after eight consecutive incorrect or non-responsive questions.

#### Complement syntax

The experimental material of complement syntax was divided into two types of sentences, the first was the sentential complement syntax, which was adapted from the “that” structure of complement syntax in the study by de Villiers and Pyers (2002). The main verb is *yiwei* (falsely think). The complement syntax test task adds a simple sentence to the material studied by de Villiers to avoid the possibility of children answering based on the last scenario.

The second type of sentence was a phrasal complement syntax, adapted from the research materials in Cheung (2006) study, in which the main verb was the mental verb “forget.” For example, *Xiaoming qu xuexiao* (Xiaoming went to school), *Xiaoming wangle dai huabi* (Xiaoming forgot to bring a paintbrush), *danshi daile qianbi* (but brought his pencil), *Xiaoming hen kaixin* (Xiaoming was very happy). Five experimental sentences were prepared for each of the two types of sentences, accompanied by corresponding pictures to facilitate participants’ comprehension.

After the experimental materials for the complement syntax were prepared, they were revised by three master’s students of Chinese language and literature to conform to the grammatical norms of Mandarin. Then, three ASD rehabilitation counselors or teachers with many years of experience judged the experimental sentences. The judgment was based on two points: first, whether the things involved in the sentences were familiar to ASD children; second, whether the sentences matched with the corresponding pictures. The sentences and pictures with low scores (less than 3) and poor consistency (less than 80%) were revised according to the principle of scoring from low to high on a scale of 1–5. They were revised until the consistency of the scorers was greater than 80%, forming the final experimental sentences.

The complement syntax test was conducted by looking at the pictures, listening to the sentences, and answering or pointing to the pictures. The following is an example based on the sentential complement syntax.

**Figure 1:**
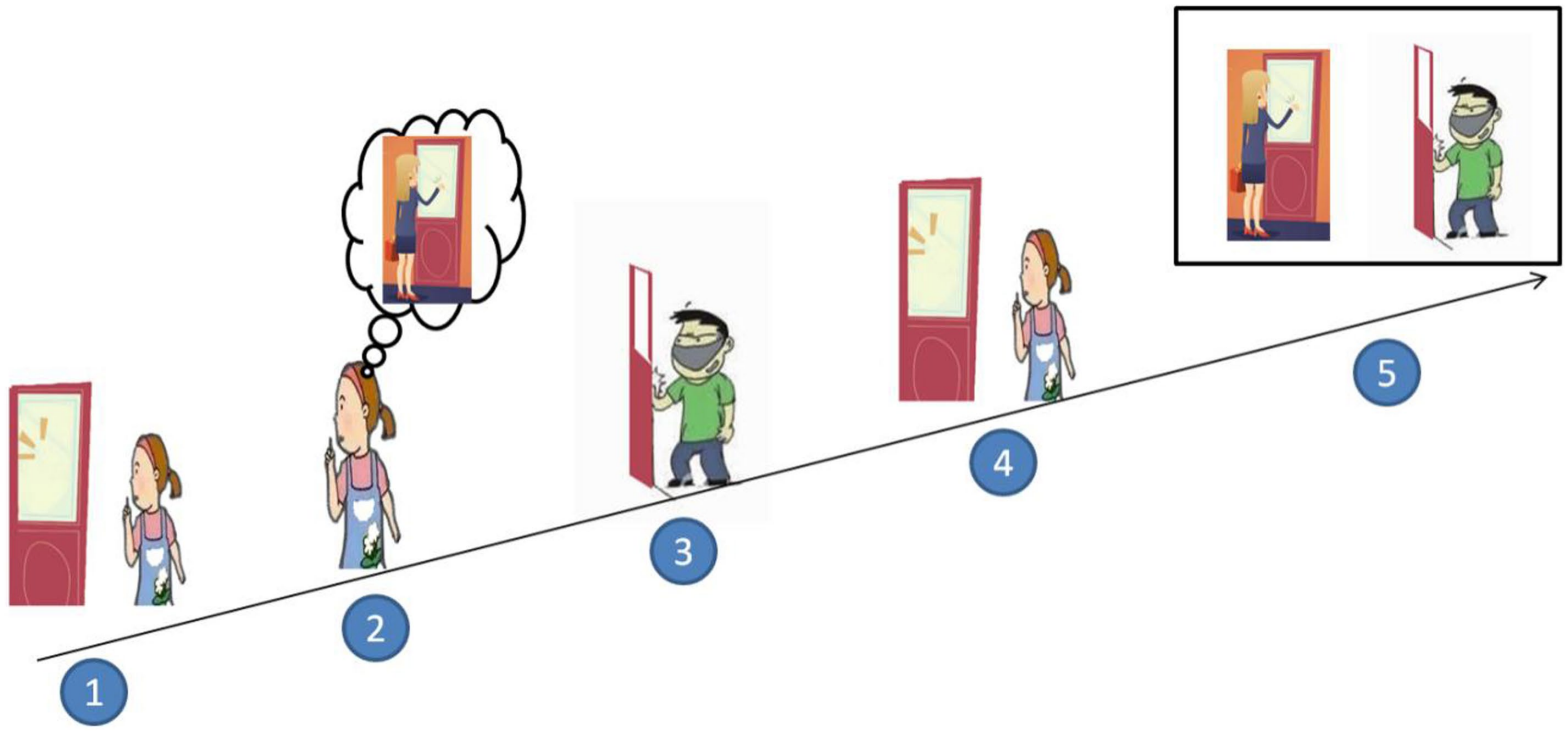
Example of sentential complement syntax.

The first picture was shown (See Figure 1) and the voice was presented with *Xiaohong tingdao you ren qiaomen* (Xiaohong heard someone knocking at the door).

The first picture disappeared and then the 2nd picture was shown, and the voice says *Xiaohong yiwei mama zai qiao men* (Xiaohong falsely thinks her mother is knocking on the door*).*

The second picture disappeared and then the third picture was presented, with the voice saying *shijishang shi huai ren zai qiaomen* (Actually, a bad guy is knocking).

The third picture disappeared and the first picture reappeared, with the voice saying.

*Xiaohong kanzhe men* (Xiaohong is looking at the door).

The first picture disappears and the second and third pictures are presented at the same time (the pictures were arranged left to the right) and the child was asked *Xiaohong yiwei shui zai qiaomen* (Who did Xiaohong think was knocking at the door?). The left–right order of the two pictures was randomized to eliminate the influence of regular responses and memory factors. Children could either answer verbally or identify the pictures with one point awarded for each correct response. The experimental procedure of phrasal complement syntax was the same as that of sentential complement syntax.

#### Verbal false belief

Two FB tasks were used to measure verbal FB using the Sally-Anne task developed by Baron-Cohen et al. (1985) and Smarties Unexpected Contents Task developed by Perner et al. (1987). Two illustrated stories were used for each task. The character names in the test were replaced with common Chinese names. For example:

##### Sally-Anne

This is Xiaogang and this is Xiaoli. Xiaogang puts the ball in the basket and goes home. When Xiaogang is not there, Xiaoli takes the ball from the basket to the box. Now Xiaogang is back. Questions: (1) Where is the ball now? (reality control question) (2) Where did Xiaogang put the ball when he went out? (memory control question) (3) Where will Xiaogang say the ball is? (test question) (4) Where did Xiaogang go first to find the ball? (test question) Children can answer the questions verbally or by pointing to the image. The test questions were asked only after the child had correctly answered the control questions. Control questions were not scored, but test questions were given one point for each correct response. Children who did not pass the control questions received a score of zero.

##### Smarties unexpected contents task

The researcher presented the children with a potato chip box (a common brand in Chinese supermarkets) and asked, “Look, what’s in this?” After they answered that it contained potato chips, the researcher said, “Let us see what’s inside.” The potato chip box was opened and the child was presented with a paintbrush. After making it clear to the children, the researcher asked (1) What is actually in the box now? (control question) (2) The first time you see this unopened box, what would you say was in the box? (test question). The researcher presented a common doll, “The little doll comes over and he does not see what is inside the box.” (3) when the doll sees the box, what would he say is in the box? (test question) Control questions were not scored. One point was given for each correct response to the test questions. Children who did not pass the control questions received a score of zero.

##### Non-verbal false belief

The nonverbal FB was based on tasks used in Baron-Cohen et al. (1986) and Durrleman et al. (2016). The mechanical I and intentional tasks were selected as experimental materials. The mechanical I task was mainly used for children to understand the test. The intentional task, which involves the attribution of mental states, was used as the non-verbal FB task and was divided into three sets of thematic stories with four pictures in each set. The researcher shuffled the four pictures, pointed to the first picture, and asked the children to “put them in order.” After the mechanical task was completed, the intentional task was conducted. Following Baron-Cohen et al.’s scoring principle, two points were awarded for correct placement of the pictures in a logical order, one point for correct placement of the last picture, and zero points for other placements. Mechanical tasks did not count.

## Results

### Children with autism

#### Simple correlations

Simple correlations were used to explore the relationship between the performance of ASD children on each task (presented in Table 1). Verbal FB was significantly correlated with nonverbal FB. Further, both were significantly correlated with nonverbal intelligence, PPVT-R, sentence comprehension, sentential complement syntax, and phrasal complement syntax. Sentential and phrasal complement syntax were significantly correlated with nonverbal intelligence, PPVT-R, and sentence comprehension.

**Table 1:** Simple correlations results for the ASD children.

	1	2	3	4	5	6	7
1. Age	1						
2. CRT	0.54^**^	1					
3. PPVT - R	0.74^***^	0.52^**^	1				
4. Sentence comprehension	0.05	0.37^*^	0.39^*^	1			
5. Verbal FB	0.24	0.38^*^	0.508^**^	0.59^**^	1		
6. Nonverbal FB	0.43^*^	0.47^**^	0.67^***^	0.60^***^	0.87^***^	1	
7. Sentential complements syntax	0.21	0.38^*^	0.54^**^	0.37^*^	0.70^***^	0.72^***^	1
8. Phrasal complements syntax	0.62^***^	0.56^**^	0.69^***^	0.38^*^	0.62^***^	0.79^***^	0.63^***^

CRT, combined Raven’s test; PPVT - R, Peabody picture vocabulary test revised. *p < 0.05, **p < 0.01, ***p < 0.001.

#### Regression for verbal false belief of autism spectrum disorder children

Regression analyses (Stepwise Regression with Forward Selection Method) were conducted to explore the unique contribution of sentential and phrasal complement syntax on verbal FB in ASD children after controlling for other variables. Previous studies revealed that age and intelligence are closely related to FB (Wellman et al., 2001; Cheung et al., 2004; Lin et al., 2015). Therefore, age and nonverbal intelligence were included as control variables in the first step of the regression model. Neither was correlated with verbal FB, *F_inc_* (2, 27) = 2.26, *p* > 0.05. Since PPVT-R and sentence comprehension were correlated with verbal FB, they were entered into the equation in the second and third steps, respectively. PPVT-R accounted for 18% of the variance in verbal FB, *F_inc_* (1, 26) = 6.91, *p* < 0.05. Subsequently, sentence comprehension accounted for 11% of the verbal FB variance, *F_inc_* (1, 25) = 5.02, *p* < 0.05. To explore the unique effects of sentential and phrasal complement syntax on the verbal FB of ASD children, controlling for the aforementioned variables, the two types of syntax were entered into the equation together in the fourth step using stepwise analysis (Tager-Flusberg and Joseph, 2005; Lin et al., 2015). Results showed that only the sentential complement syntax significantly predicted verbal FB, *F_inc_* (1, 24) = 13.64, *p* < 0.01, accounting for 19% of the variance (see Table 2).

**Table 2:** Regression analysis results of verbal FB for the ASD children.

Variable	*B*	*SE B*	β	*t*	R	R2	ΔR2	ΔF
Step 1								
Age	0.01	0.03	0.05	0.22	0.38	0.14	0.14	2.26
CRT	0.10	0.06	0.35	1.67				
Step 2								
PPVT - R	0.07	0.03	0.64	2.63*	0.57	0.32	0.18	6.91*
Step 3								
Sentence comprehension	0.72	0.32	0.42	2.24*	0.66	0.44	0.11	5.02*
Step 4								
Sentential complements syntax	1.34	0.38	0.57	3.56*	0.79	0.63	0.19	12.64**

CRT, combined Raven’s test; PPVT - R, Peabody picture vocabulary test revised. *p < 0.05, **p < 0.01.

#### Regression for nonverbal false belief of autism spectrum disorder children

Verbal FB and nonverbal FB have different requirements for the language abilities of ASD children. Thus, the relationship between different types of complementary syntax and nonverbal FB in Mandarin-speaking children with autism was further explored. Using the same regression analysis model, age, nonverbal intelligence, PPVT-R, and sentence comprehension were entered into the equation in steps 1, 2, and 3, respectively, and sentential and phrasal complement syntax were entered into the last step of the equation. After controlling for age and nonverbal intelligence, PPVT-R accounted for 22% of the nonverbal FB variance, *F_inc_* (1, 26) = 11.30, *p* < 0.001, sentence comprehension explained 10% of the nonverbal FB variance, *F_inc_* (1, 25) = 6.20, *p* < 0.05. After controlling for these variables, sentential and phrasal complement syntax significantly predicted nonverbal FB. The phrasal complement syntax accounted for 15% of the variance, *F_inc_* (1, 24) = 14.27, *p* < 0.001, while the sentential complement syntax only accounted for 5%, *F_inc_* (1, 23) = 5.00, *p* < 0.05 (see Table 3).

**Table 3:** Regression analysis results of nonverbal FB for the ASD children.

Variable	*B*	*SE B*	β	*t*	*R*	*R* ^2^	Δ*R*^2^	Δ*F*
Step 1								
Age	0.02	0.02	0.25	1.29	0.52	0.27	0.27	4.93*
CRT	0.06	0.04	0.34	1.72				
Step 2								
PPVT - R	0.05	0.02	0.71	3.36**	0.70	0.49	0.22	11.30**
Step 3								
Sentence comprehension	0.48	0.19	0.40	2.49*	0.77	0.59	0.10	6.20*
Step 4								
Phrasal complements syntax	0.93	0.25	0.59	3.78*	0.86	0.74	0.15	14.27**
Step 5								
Sentential complements syntax	0.54	0.24	0.33	2.24*	0.89	0.79	0.05	5.00*

CRT, combined Raven’s test; PPVT - R, Peabody picture vocabulary test revised. *p < 0.05, **p < 0.01.

### Typically developing children

#### Simple correlations

The results of correlational analyses for the TD children matched on language ability of the ASD children are shown in Table 4. Verbal FB was significantly and positively correlated with nonverbal FB, and both were significantly correlated with age, nonverbal intelligence, PPVT-R, sentence comprehension, and sentential complement syntax. Sentential complement syntax was significantly correlated with phrasal complement syntax, and they were both significantly correlated with age, word comprehension, and sentence comprehension. Sentential complement syntax was significantly correlated with nonverbal intelligence and nonverbal FB, while phrasal complement syntax was not correlated with either. Furthermore, age showed high correlations with nonverbal intelligence and PPVT-R.

**Table 4:** Simple correlations results for the TD children.

	1	2	3	4	5	6	7
1. Age	1						
2. CRT	0.90^***^	1					
3. PPVT-R	0.98^***^	0.91^***^	1				
4. Sentence comprehension	0.57^**^	0.39^*^	0.53^**^	1			
5. Verbal FB	0.65^***^	0.50^**^	0.68^***^	0.64^***^	1		
6. Nonverbal FB	0.54^**^	0.42^*^	0.52^**^	0.37^*^	0.46^*^	1	
7. Sentential complements syntax	0.74^***^	0.61^***^	0.75^***^	0.68^***^	0.86^***^	0.56^***^	1
8. Phrasal complements syntax	0.38^*^	0.34	0.40^*^	0.49^**^	0.62^***^	0.35	0.67^***^

CRT, combined Raven’s test; PPVT - R, Peabody picture vocabulary test revised. *p < 0.05, **p < 0.01, ***p < 0.001.

#### Regression for verbal false belief of typically developing children

A similar analysis approach was applied for language-matched TD children as ASD children to explore the relationship between complement syntax and verbal FB. Correlation analyses showed that the age of TD children was highly correlated with nonverbal intelligence. When age and nonverbal intelligence were entered into the equation together, the equation showed multicollinearity. Based on the principles of regression analysis for multiple cointegrations and the purpose of this study, the regression hypothesis was re-tested after age was excluded. The results showed that the maximum variance inflation factor of each variable in the regression equation was less than 10, and the problem of multicollinearity in the equation disappeared. The other hypotheses also met the requirements of the multiple linear regression analysis. Thus, nonverbal intelligence, PPVT-R, and sentence comprehension entered the equation in steps 1, 2, and 3, respectively. Finally, sentential and phrasal complement syntax entered the equation together.

The results showed that PPVT-R accounted for 25% of the TD children’ verbal FB variance, *F_inc_* (1, 27) = 15.60, *p* < 0.001. After controlling for nonverbal intelligence and PPVT-R, sentence comprehension accounted for 7% of the variance, *F_inc_* (1, 26) = 4.88, *p* < 0.05. After excluding the above variables, only sentential complement syntax significantly predicted verbal FB, *F_inc_* (1, 25) = 17.26, *p* < 0.001, accounting for 16% of the variance (see Table 5).

**Table 5:** Regression analysis results of verbal FB for the TD children.

Variable	*B*	*SE B*	β	*t*	*R*	*R* ^2^	Δ*R*^2^	Δ*F*
Step 1								
CRT	0.13	0.04	0.50	3.09**	0.50	0.25	0.25	9.55***
Step 2								
PPVT-R	0.08	0.02	1.27	3.95**	0.73	0.53	0.27	15.60***
Step 3								
Sentence comprehension	0.29	0.13	0.33	2.21*	0.78	0.60	0.07	4.88*
Step 4								
Sentential complements syntax	1.39	0.33	0.71	4.15***	0.87	0.76	0.16	17.26***

CRT, combined Raven’s test; PPVT - R, Peabody picture vocabulary test revised. *p < 0.05, **p < 0.01,***p < 0.001.

#### Regression for nonverbal false belief of typically developing children

In the regression analysis of nonverbal FB in TD children, after controlling for nonverbal ability, PPVT-R, and sentence comprehension, sentential and phrasal complement syntax failed to predict nonverbal FB (see Table 6).

**Table 6:** Regression analysis results of nonverbal FB for the TD children.

Variable	*B*	*SE B*	β	*t*	*R*	*R* ^2^	Δ*R*^2^	Δ*F*
Step 1								
CRT	0.1	0.04	0.42	2.43*	0.42	0.17	0.17	5.91*
Step 2								
PPVT-R	0.05	0.03	0.81	2	0.53	0.28	0.11	4.16
Step 3								
Sentence comprehension	0.09	0.17	0.1	0.5	0.54	0.29	0.01	0.25

CRT, combined Raven’s test; PPVT - R, Peabody picture vocabulary test revised. *p < 0.05.

## Discussion

This study explored the relationship between complement syntax and FB in Mandarin-speaking children with autism, and the characteristics of this relationship with TD children. The findings suggest that complement syntax independently predicts FB in ASD children after controlling for other variables. However, this predictive effect varies according to the type of complement syntax and the linguistic requirements of the FB task. TD children’s sentential complement syntax predicted only verbal FB.

### The relationship between different types of complement syntax and false belief

Results demonstrated that after controlling for age, nonverbal intelligence, PPVT-R, and sentence comprehension, sentential complement syntax predicted verbal and nonverbal FB in children with autism. This is consistent with the results of Durrleman et al. (2017). After controlling for nonverbal intelligence, Durrleman et al. (2017) found that complement syntax was positively associated with verbal FB in ASD children. Similar results were obtained for TD children matched for linguistic ability in the present study, with sentential complement syntax independently predicting verbal FB. This is consistent with the findings of de Villiers and Pyers (2002) in an English-speaking population. In a study exploring the relationship between complement syntax and theory of mind in Mandarin-speaking TD children, Mo and Su (2009) also found that complement syntax significantly predicted FB when controlling for semantic comprehension and working memory. Although Cheung et al. (2004) did not find a predictive effect of sentential complement syntax on verbal FB in English-and Cantonese-speaking children, the two were significantly correlated. The sentential complement syntax embeds independent propositions into another sentence, and this meta-representation-like approach helps children understand the propositions. Therefore, it provides a convenient representation for children to understand the psychology of others. This representational approach is also applicable to understanding nonverbal FB. The results of the present study are similar to those of Durrleman et al. (2016). Even after controlling for variables such as nonverbal cognitive abilities, the sentential complement syntax was still significantly associated with nonverbal FB in ASD children. Some researchers have found similar results in the relationship between complement syntax and nonverbal FB in children with hearing impairment (Schick et al., 2007). These findings further support the theoretical view proposed by de Villiers et al. (2014) that complement syntax contributes to the representational theory of mind in children with special conditions, such as autism.

In addition to sentential complement syntax, phrasal complement syntax also independently predicted nonverbal FB in ASD children but failed to predict verbal FB. This indicates that ASD children could comprehend nonverbal FB through phrasal and sentential complement syntax, whereas children with autism could only characterize FB through sentential complement syntax in a highly linguistically demanding task. This further confirms that the linguistic demands of FB tasks affect children’s understanding and representation of FB (Chandler et al., 1989; Miller, 2004). Furthermore, phrasal complement syntax explained 15% of the nonverbal FB variance in ASD children and sentential complement syntax only accounted for 5% of the variance. In the verbal FB task, sentential complement syntax accounted for 19% of the variance, while phrase structure complement syntax accounted for none. There are two possible reasons for this. First, in terms of syntactic complexity, the phrasal complement syntax has a simpler syntactic structure with the verb phrase as the structural object of the sentence. In contrast, the sentential complement syntax takes a complete sentence as the object of the main sentence, and the syntactic structure is relatively complex. ASD children may be more likely to activate phrasal complement syntax during simple tasks. When dealing with more linguistically demanding tasks, phrasal complement syntax may not provide effective representations, requiring a more complex structure of sentential complement syntax to complete the task. Second, from the perspective of subject switching, phrasal complement syntax has only one subject, while sentential complement syntax has two independent subjects. Children need to distinguish the subject of the sentential complement syntax when processing for comprehension. This subject-role distinction may help children to think from different subject perspectives. The above results suggest that the relationship between different types of Chinese complement syntax and the FB of children with autism is influenced by linguistic difficulty in the theory of mind task. When the linguistic requirements of the FB task were reduced, ASD children could comprehend the FB using phrasal and sentential complement syntax.

### Differences in the relationship between complement syntax and false belief in autism spectrum disorder and typically developing children

TD children had similar patterns to ASD children in the complement syntax-verbal FB relationship. However, the two groups of children showed differences in their relationship with nonverbal FB. Complement syntax failed to predict nonverbal FB in TD children. This is consistent with the results of the Poltrock (2010) study, where after controlling for age, sentential complement syntax was no longer associated with nonverbal FB in TD children. Similar results were obtained in the longitudinal study, where De Mulder et al. (2019) found that first-year sentential complement syntax failed to predict second-year nonverbal FB. This indicates, to some extent, that TD children’s FB comprehension representations are related to nonverbal factors, such as age.

Tager-Flusberg and Sullivan (2000) argued that TD children can represent the theory of mind through social perceptual and social cognitive components. Previous research has shown that TD children can understand representations of nonverbal FB through implicit processing, such as joint attention, eye gaze, and representations of explicit verbal FB with the help of implicit FB representational mechanisms, executive functions, general language skills, and complement syntax (Surian et al., 2007; Wang et al., 2012). In contrast, children with autism have impairments in facial recognition, perception of others’ voices, and body posture (Baron-Cohen et al., 2001), and are unable to represent theory of mind through social perceptual channels. Individuals with autism who pass a theory of mind task tend to have high language abilities (Kleinman et al., 2001; Low, 2010). Tager-Flusberg and Joseph (2005) suggest that these patients decode and represent theory of mind through social cognitive components such as language, thus compensating for the deficit of social perception in theory of mind representation. Senju et al. (2009) examined theory of mind in adults with Asperger’s and found that these adults could pass a verbal form of extrapersonal theory of mind tasks, but not the nonverbal implicit theory of mind tasks. They argue that individuals with Asperger’s represent the theory of mind through language and other means. In the present study, not only did sentential complement syntax independently predict verbal as well as nonverbal FB in ASD children, but word comprehension and sentence comprehension also predicted FB; whereas TD children with equivalent language ability showed prediction only in the verbal FB task. This provides empirical evidence for Tager-Flusberg’s social cognitive component theory in a Mandarin-speaking population.

### Limitations

Although the results of the present study revealed relationships between different types of complement syntax and FB in Mandarin-speaking ASD children, there are still some limitations that need to be noted. First, the present study was a cross-sectional study and could not demonstrate the causal relationship between different types of complement syntax and FB. Thus, future studies should use longitudinal or intervention studies to explore this causal relationship. Second, some studies show that the relationship between complement syntax and FB is affected by other variables, such as executive function (Durrleman and Franck, 2015). This study did not explore these additional relationships. Therefore, future studies should explore the relationship between different types of complement syntax, executive function, and FB. Third, the verbs of the complement syntax used in this study were psychological. Some researchers suggest that word type affects FB (Lin et al., 2015), so this should be considered for future studies. Finally, The present study explored the relationship between complement syntax and theory of mind in children with ASD from an episodic behavioral perspective. However, the neurocognitive processing of both is uncertain. Recent studies have shown that EEG graph-theoretic measures could be used to diagnose children with ASD and reveal social cognitive processes in the brains of children with ASD (Tanu and Kakkar, 2019; Wadhera and Kakkar, 2021). Thus, future studies could use EEG graph-theoretic measures to explore the influence of complement syntax on the theory of mind of children with ASD and reveal its neurocognitive mechanisms.

### Conclusion

Complement syntax can predict FB in children with autism. As the language requirement of the FB task decreased, ASD children used two kinds of complement syntax for their predictions. For the verbal FB task, ASD children could only predict FB using the more complex structure of sentential complement syntax. Under the nonverbal FB task, FB can be predicted by phrasal and sentential complement syntax. There are different representations in the understanding of FB between ASD and TD children. ASD children represent verbal and nonverbal FB through language abilities, such as complement syntax. In contrast, TD children use sentential complement syntax to represent FB in verbal tasks.

## Data availability statement

The original contributions presented in the study are included in the article/supplementary material, further inquiries can be directed to the corresponding authors.

## Ethics statement

The studies involving human participants were reviewed and approved by the Human Ethics Committee of Zhejiang Normal University. Written informed consent to participate in this study was provided by the participants’ legal guardian/next of kin.

## Author contributions

QG designed this study, performed the experiments, and authored and reviewed the manuscript. QP authored and reviewed drafts of the manuscript. QL designed the experiments. TW, SC, and YL reviewed the manuscript. BH designed the experiments and reviewed drafts of the paper. All authors contributed to the article and approved the submitted version.

## Funding

This work was supported by the Zhejiang Normal University Young Doctor Dedicated project (Grant no. ZZ323205020520013083 to QG), the National Key Research and Development Program of China (Grant no. 2022YFC2705201 to QL), the National Social Science Fund of China (Grant no. 21&ZD293 to TW), the Social Science Fund of Shaanxi in China (Grant no. 2022P025 to TW), and the Social Science Fund of Xi’ an in China (Grant no. 22JY22 to TW).

## Conflict of interest

BH was employed by Zhejiang Bema Postdoctoral Workstation.

The remaining authors declare that the research was conducted in the absence of any commercial or financial relationships that could be construed as a potential conflict of interest.

## Publisher’s note

All claims expressed in this article are solely those of the authors and do not necessarily represent those of their affiliated organizations, or those of the publisher, the editors and the reviewers. Any product that may be evaluated in this article, or claim that may be made by its manufacturer, is not guaranteed or endorsed by the publisher.

## Footnotes

^1^The prevalence of autism is higher in males than in females, and studies have not found a significant effect of gender on the theory of mind and complement syntax in ASD children. The present study did not control for gender when selecting subjects with autism based on the results of existing studies.
